# Droplet digital PCR (ddPCR) for the detection and quantification of *Ureaplasma* spp.

**DOI:** 10.1186/s12879-021-06355-6

**Published:** 2021-08-11

**Authors:** Yanfang Huang, Huifen Pan, Xiaoqin Xu, Panpan Lv, Xinxin Wang, Zhen Zhao

**Affiliations:** 1 Clinical Laboratory, Minhang Hospital, Fudan University, No. 170, Xinsong Road, Shanghai, China; 2 Department of Molecular Medicine, Biomed-Union Co. Ltd. Shanghai, Shanghai, China

**Keywords:** *Ureaplasma parvum*, *Ureaplasma urealyticum*, qPCR, Droplet digital PCR, Absolute quantitation, Cervicitis

## Abstract

**Background:**

*Ureaplasma* spp*.* are associated with various infectious diseases in females, but there is still limited evidence regarding whether they are related to nonspecific cervicitis. The aim of this study was to develop and evaluate a digital droplet PCR (ddPCR) assay for the detection and quantification of *Ureaplasma* spp. in cervical swabs.

**Methods:**

A total of 267 non-specific cervicitis (NSC) patients and 195 asymptomatic females were included in this study. We produced standard curves for *Ureaplasma* spp*.* to evaluate the analytical performance of the ddPCR assay. Then, we detected and quantified the bacterial load of *Ureaplasma* spp. in cervical swabs.

**Results:**

The prevalences of *U. parvum* were 37.8% (101/267) and 29.7% (58/195),  *U. urealyticum* were 9.0% (24/267) and 8.7% (17/195) in the NSC group and control group, respectively. In addition, the median copy number of *U. parvum* was 2.5 × 10^4^ copies/ml (*n* = 101) in the NSC group and 9.2 × 10^3^ copies/ml (*n* = 58) in the control group. The *U. parvum* load in the NSC group was significantly higher than that in the asymptomatic individuals (*P* < 0.001). whereas the median load of *U. urealyticum* was 8.4 × 10^3^ copies/ml (*n* = 24) and 1.4 × 10^3^ (*n* = 17) copies/ml in the two groups, respectively, , the difference was not statistically significant (*P* = 0.450).

**Conclusions:**

Our study is the first to develop a droplet digital PCR (ddPCR) method for the detection and quantification of *Ureaplasma* spp. in clinical samples, and the method has excellent analytical performance and a wide range of clinical application prospects.

**Supplementary Information:**

The online version contains supplementary material available at 10.1186/s12879-021-06355-6.

## Introduction

*Ureaplasma* spp. has 14 known serotypes that were divided into two biovars: *Ureaplasma parvum and Ureaplasma urealyticum*. This pathogen is suspected to be the causative agents of non-gonococcal urethritis [1], bacterial vaginosis [2], cervicitis [3], and multiple adverse pregnancy outcomes such as spontaneous preterm birth [4], chorioamnionitis [5], and preterm premature rupture of membranes (PPROM) [6]. In addition, some case reports have described rare invasive infections from *Ureaplasma* spp., such as idiopathic hyperammonemia [7], spontaneous bacterial pericarditis [8], multifocal abscesses [9], and osteomyelitis [10], especially in immunocompromised patients.

However, the roles of *U. parvum* and *U. urealyticum* are sometimes controversial. A meta-analysis included seven eligible case-control studies, including 1507 NGU patients and 1223 controls. The results suggested that *U. urealyticum* was significantly more prevalent inNGU patients, but *U. parvum* was found more oftenin controls [11]. Nevertheless, several clinical and fundamental types of research havereported that *U. parvum* induces adverse outcomes by causing a severe inflammatory response [4, 12, 13]. The controversy has been well explainedby Viscardi RM et al. [14], attributing it partly due to the presence of potential confounding factors in specimens, such as *U. parvum* and *U. urealyticum* based on culture or PCR assays. These reasons make the interpretation of many previous studies extremely difficult. Moreover, some scholars have proposed that the host genetic background and bacteria load have an impacte on the disease outcomes with *Ureaplasma* spp. [15, 16].

Of note, The European STI Guidelines Editorial Board issued a position statement that only people with a high *Ureaplasma* spp. load should be considered for treatment [17]. The quantitative bacterial load in all previous studies has used qPCR technology with external references [18, 19]; undoubtedly, it is a challengingto generatea standard curve. Digital droplet PCR (ddPCR) is a method of absolute nucleic acid quantification without external references and robustness to variations in PCR efficiency [20, 21]. Several studies have showed that ddPCR has much higher sensitivity and is more resistant to PCR inhibition than qPCR [22, 23]. A recent study suggested that the copy numbers obtained by qPCR were 1 to 31 times higher than those obtained byddPCR (mean: 9.7; SD 7.7) [24], indicating that ddPCR has more accurate quantitative capabilities than qPCR.

Hence, the purpose of this study was to develop ddPCR for the detection and accurate quantification of *Ureaplasma* spp. in non-specific cervicitis patients and an asymptomatic physical examination population (control group).

## Materials and methods

### Study population

Patients attending the gynaecology outpatient or health clinic in Minhang Hospital, Fudan University were recruited for this study from July 2019 to November 2020. All women 1) were aged ≥18 years without co-infection with HIV or autoimmune diseases, 2) had exhibited symptoms of cervical discharge (purulent or mucopurulent endocervical exudate) or cervical bleeding [25], 3) had not received the gynaecologic intervention or antibiotics treatment within the preceding 3 months, 4) had not used an intrauterine contraceptive device and had been sexually active in last 3 months, 5) and were not currently pregnant or menstruating.

A total of 537 cervical samples were obtained according to protocols performed by experienced clinicians. All specimens were examined microscopically to rule out fungi and *Trichomonas vaginalis* and then subjected to HPV, *C. trachomatis*, *N. gonorrhoea*, *M. hominis, and M. genitalium*, *U. parvum*, and *U. urealyticum* testing by qPCR in septuplicate. Finally, a total of 462 samples were ultimately enrolled in this study, including 267 non-specific cervicitis patients [26, 27] and 195 asymptomatic individuals.

### Specimen collection and DNA extraction

Total DNA from 200 μl fresh cervical swab samples was extracted using MiniBest Universal Genomic DNA Extraction Kit (Takara) following the manufacturer’s instructions. The extracts were resuspended in 100 μl of nuclease-free water and  stored at − 20 °C or − 80 °C until use. The concentration of nucleic acids was determined with a NanoDrop 2000 Spectrophotometer (Thermo Scientific, USA). Human papillomavirus (HPV), *C. trachomatis*, *N. gonorrhoea*, *M. hominis, and M. genitalium* were confirmed by commercial kits based on TaqMan probes (Shanghai Kehua Bio-Engineering, or ZJ Bio-Tech Co., Ltd., Shanghai, China) according to the manufacturer’s instructions. *U. parvum* serovar 1 (ATCC 27813) and *U. urealyticum* serovar 4 (ATCC 27816) were used as standard strains.

### Real-time quantitative (qPCR)

QPCR amplification was performed in a total volume of 20 μl, including the 10 μl of Probe qPCR Mix (with UNG) (Takara), 500 nM target/reference primers, 250 nM target/reference probes, and 9 μl of each template DNA. The qPCR conditions were as follows: 25 °C for 10 min, followed by 45 cycles of 15 s at 95 °C and 34 s at 60 °C. Fluorescent accumulation data were analysed using ABI 7500 Software Version 2.3 (Applied Biosystems). Threshold cycle (CT) values of < 40 were defined as a positive result for the *U. parvum* and *U. urealyticum*, respectively. In addition, the quality of PCR extraction was monitored by amplifying human glyceraldehyde-3-phosphate dehydrogenase (GAPDH) in cervical swabs. CT values of > 37 suggest the failure of DNA extraction or qPCR amplification. Standard curves were generated by plotting the CT of the qPCR performed on a ten fold dilution series of purified DNA from *U. parvum* *and U. urealyticum*.

The primers and probes were designed based on the ParC gene conserved regions of *U. parvum* and *U. urealyticum*, respectively (Additional file 1), which have previously been used for qPCR [28].

*U. parvum*, forward: 5′-TAGTTGCTCATAAAATCAC-3′,

Reverse: 5′-CGTTCCATATATAAACAGCTATAAC-3′,

Probe: 5′- FAM-CTATGCGTGAAAAGATG-BHQ1–3′;

*U. urealyticum*, forward: 5′- TAAGTGTTTTAGTATTAGTGAGC-3′;

Reverse: 5′- TGCTGCTAAAACGCTTTGTGC-3’C;

Probe: 5′- FAM-CACCATCACCACTTTATT-BHQ1–3′.

### Digital droplet PCR (ddPCR)

The same primers and probes were used for ddPCR reactions as qPCR. ddPCR was performed using a QX200 Droplet Digital PCR system (Bio-Rad). The Mastermix for ddPCR included 1× ddPCR Supermix for Probes (no dUTP), 900 nM target/reference primers, 250 nM target/reference probes, and 9 μl sample DNA. The generation of droplets was performed by the QX200 Droplet Generator according to the manufacturer’s protocols. PCR amplification was carried out using an Applied Biosystems Veriti 96-Well Thermal Cycler with the following PCR conditions: 95 °C for 10 min followed by 40 cycles of denaturation at 94 °C for 30 s, 60 °C for 1 min and the enzyme was deactivated at 98 °C for 10 min. The plate was stored at 4 °C until the droplets were analysed with QX200 Droplet Reader and QuantaSoft software version 1.7.4 (Bio-Rad). The threshold between positive and negative droplet populations was set manually using per-plate positive and no-template controls as a guide.

### Diagnostic performance of ddPCR for *Ureaplasma* spp.

First, the specificity of the primers and probes for *U. parvum* and *U. urealyticum* were evaluated with 12 kinds of common microorganisms of the genitourinary tract, including *M. hominis*, *M. genitalium*, *C. trachomatis*, Human papillomavirus (HPV), Herpes simplex virus (HSV), *N. gonorrhoea, S. agalactiae, S. aureus, E. faecium, E. coli, P. aeruginosa, C. albicans.* Second, the linear dynamic range of the ddPCR assay was assessed using the serial 10-fold dilutions of the standard DNA containing the target region as described previously [18]. Each dilution in the series was tested in three technical replicate wells. DNase/RNase-free water was used as the negative control (NC). The analytical limit of the assay was determined to be the concentration of the maximum dilution factor of the series in which all 10 replicates tested positive. To compare the dynamic range of ddPCR and qPCR, 10-fold serial dilutions of *U. parvum* and *U. urealyticum* were tested using the same primers/probe sets for both ddPCR and qPCR. Octuplicate results were used for intra-assay and inter-assay calculations.

### Statistical analysis

Continuous variables are expressed as “the mean  ±  standard deviation”. Intra- and interassay coefficients of variability (CV) were calculated for methodological evaluation. Continuous variables between the two groups were compared using Student's t-*test. *The medians were compared using Wilcoxon rank sum test. The Pearson correlation coefficient was performed to estimate the correlation betweenqPCR and ddPCR results of clinical samples. A *P-*value < 0.05 was considered a significant difference. The data were calculated in Stata 13.0 (StataCorp, TX, USA) and plots of linear regression were generated with GraphPad Prism 8.0 Software, Inc. 

## Results

### Analytical performance of the ddPCR assay

**Fig. 1:**
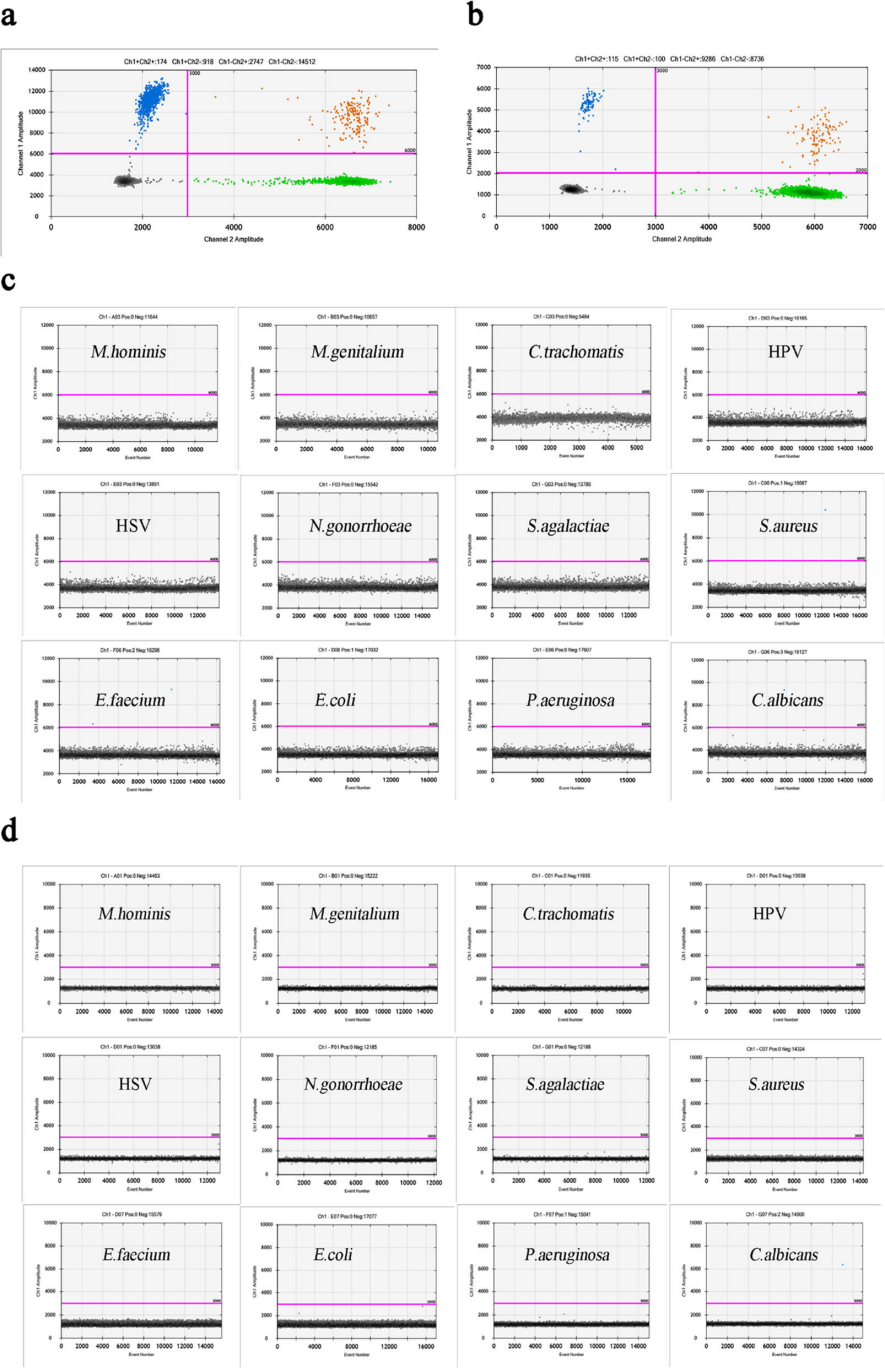
Specificity of Primers and probes for *U. parvum* and *U. urealyticum*. **a** Positive droplets of *U. parvum* with channel amplitude signals above 6000. **b** Positive droplets of *U. urealyticum* with channel amplitude signals above 2000. **c** and **d** The primers and probes for *U. parvum* (c) and *U. urealyticum* (d) showed excellent specificity, without cross-reaction to 12 kinds of common microorganisms of the genitourinary tract, including *M. hominis*, *M.genitalium*, *C. trachomatis*, Human papilloma virus, Herpes simplex virus, *N. gonorrhoeae, S.agalactiae, S. aureus, E. faecium, E. coli, P. aeruginosa, and C. albicans*

We found positive droplets of *U. parvum* and *U. urealyticum* with channel amplitude signals above 6000 and 2000, respectively (Fig. 1). The ddPCR assay showed excellent specificity with no cross-reaction with twelve kinds of urogenital microorganisms. In addition, the ddPCR assay exhibited excellent intra- and inter assay coefficients of variability (CV). ddPCR has a lower intra-assay CV (0.36–0.42%) and a lower inter-assay CV (0.78–1.59%) for *U. parvum*, as well as a lower intra-assay CV (0.31–0.81%) and a lower inter-assay CV (1.43–1.48%) for the *U. urealyticum,* respectively (Table 1).

**Table 1:** The diagnostic performance of ddPCR for *Ureaplasma* spp.

Analytical performance	*U. parvum*	*U. urealyticum*
Specificity (%)	100	100
Liner range (copies/μl)	3.20 ~ 3200	1.50–1500
The limit of detection	0.64	1.1
Intra-assay CV (%)	0.36–0.42	0.31–0.81
Inter-assay CV (%)	0.78–1.59	1.43–1.48

**Fig. 2:**
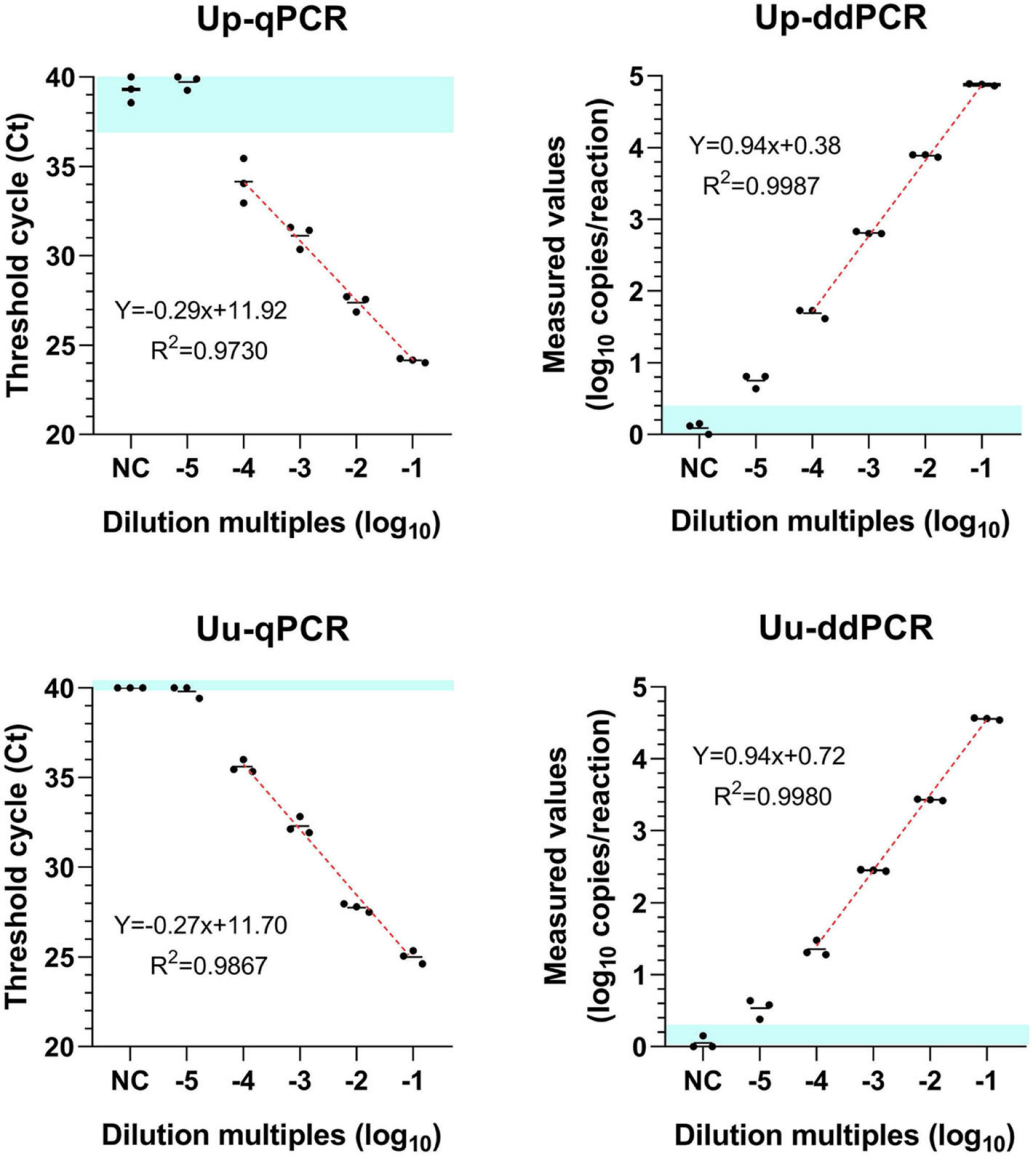
Comparison of the dynamic range of ddPCR and qPCR. A plot of the results from a linearity experiment to compare the correlation coefficient of ddPCR and qPCR. Dilution multiples are plotted on the X-axis measured values of qPCR (**A** and **C**) and ddPCR (converted to log_10_) are plotted on the Y-axis using GraphPad Prism 8.0. The data represent three independent experiments, with each concentration repeated 3 times (mean ± SD). Linear regression of the ddPCR assay for ten fold serially diluted DNA of *Ureaplasma* spp.

As shown in Fig. 2, the liner range of ddPCR was 3.2–3200 copies/μl with R^2^ = 0.9987 for *U. parvum* and was 1.5–1500 copies/μl with R^2^ = 0.9980 for *U. urealyticum,* respectively. The limit of detection of qPCR is ten times for that of ddPCR for *U. parvum* and *U. urealyticum *(Fig. 2).

### Prevalence of *Ureaplasma* spp. in the NSC and control groups

**Fig. 3:**
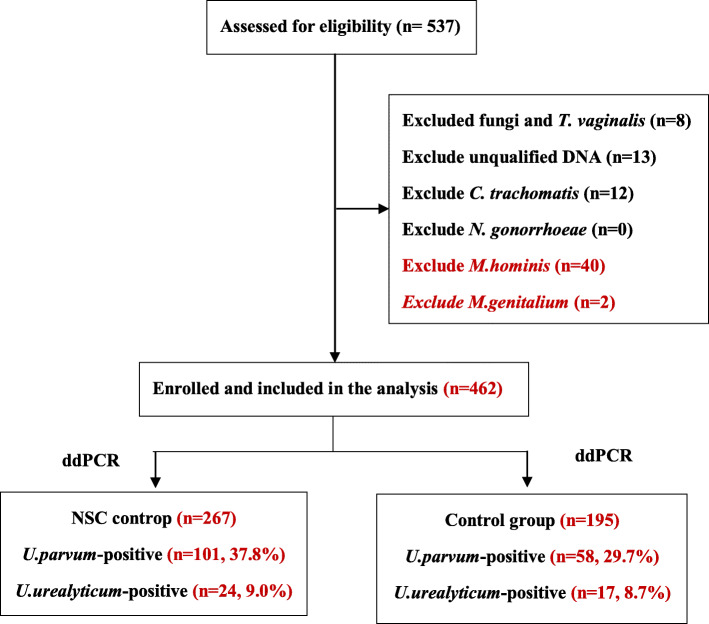
Schematic flow chart showing the inclusion specimens in this study

There were 267 females with non-specific cervicitis in the case group, with a mean age of 40.56 (range 20–74 years; SD ± 11.06 years), and 195 asymptomatic females in the control group, with a mean age of 43.37 (range 23–68 years; SD ± 8.03 years). There were no differences in age between the two groups (*P* > 0.05). The prevalence of *U. parvum* was 37.8% (101/267) in the NSC group and 29.7% (58/195) in the control group, respectively. The prevalence of *U. urealyticum* was  9.0%24/267) in the NSC group and 8.7%17/195) in the control group (Fig. 3).

### Correlation analysis of ddPCR and qPCR

**Fig. 4:**
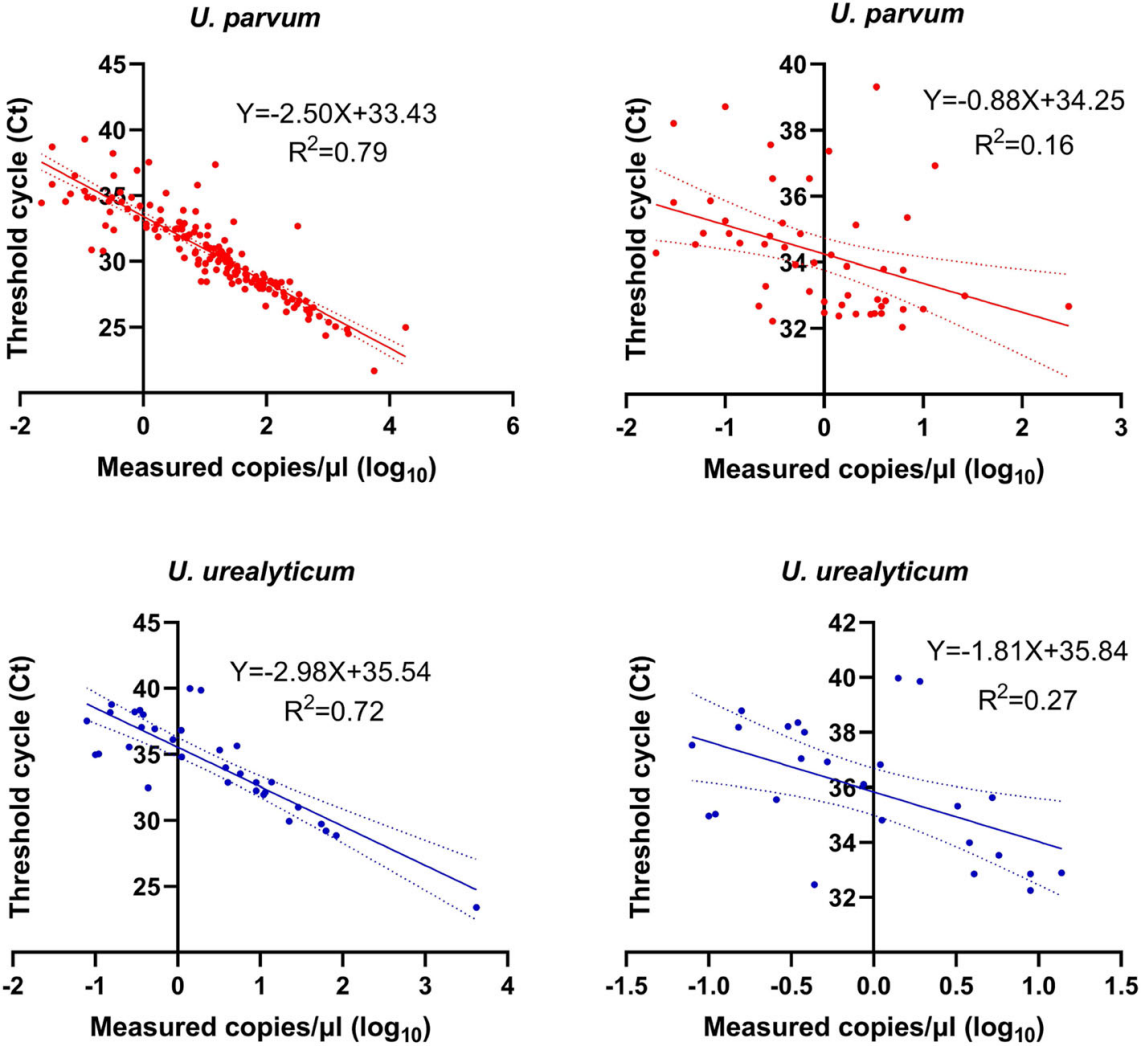
Correlation of *U. parvum* and *U. urealyticum* between qPCR and ddPCR assays. Correlation and regression analysis of ddPCR and qPCR. Measured copies are plotted on the X-axis (converted to log_10_) and Ct values of qPCR on the Y-axis using GraphPad Prism 8.0

*U. parvum* and *U. urealyticum* positive specimens were tested by ddPCR and qPCR simultaneously. The correlation coefficients between ddPCR and qPCR were 0.79 and 0.72 for *U. parvum and U. urealyticum*, respectively. However, it is worth noting that the Spearman correlation coefficient was very low when the Ct value was greater than 32 (R^2^ = 0.16 for *U. parvum and* R^2^ = 0.27 for *U. urealyticum*) (Fig. 4).

### Absolute quantitation of *U. parvum* and *U. urealyticum*

Based on ddPCR, the median bacterial load for *U. parvum* was 2.5 × 10^4^ copies/ml in the NSC group (*n* = 101) and 9.2 × 10^3^ copies/ml in the control group (*n* = 58). However, the median load for *U. urealyticum* was 8.44 × 10^3^ copies/ml in the NSC group (*n* = 24) and 1.38 × 10^3^ copies/ml in the control group (*n* =17 ) (Table 2). The load of *U. parvum* was significantly different between the NSC and control groups, whereas, the load of *U. urealyticum* was not significantly different between the two groups (*P* > 0.05).

**Table 2:** Copy numbers of *U. parvum* and *U. urealyticum* by ddPCR in the NSC and control groups

Group	*U. parvum*			*U. urealyticum*		
	N	Median copies/ml (IQR)	*P*	N	Median copies/ml (IQR)	*P*
NSC	101	2.5 × 10^4^ (6.0 × 10^3^ -1.1 × 10^5^)	0.000	19	8.4 × 10^3^ (1.2 × 10^3^ -3.1 × 10^4^)	0.450
Control	58	9.2 × 10^3^ (5.9 × 10^2^ -3.4 × 10^4^)		14	1.4 × 10^3^ (5.2 × 10^2^–2.5 × 10^4^)	

## Discussion

The purpose of this study was to develop a ddPCR assay for the detection and quantification of *Ureaplasma* spp.. According to the standard curve of the ddPCR assay for DNA from *ATCC* reference strains of *Ureaplasma* spp., the assays showed excellent analytical performance. Our data suggested that the precision of ddPCR is higher than that of qPCR, especially for the low-copy specimens, similar to the report of Mahendran P[29]. It can contribute to the results that the correlation of *Ureaplasma* spp. was higher between ddPCR and qPCR methods, (Fig. 4) but very poor when the Ct value was larger than 32 in clinical samples. Thus, ddPCR would more accurately quantify the load of *Ureaplasma* spp. than qPCR assay, Nevertheless, the *Ureaplasma* spp. load was assessed by qPCR assay in previous studies [18, 30]. According to Table 2, the absolutely copy number in the non-specific cervicitis group was higher than that in the control group (*P* = 0.003). In contrast, the difference in *U. urealyticum* copy number between the two groups was not statistically significant (*P* = 0.450).

*Ureaplasma* spp. can be isolated from blood, amniotic fluid, cerebrospinal fluid, sputum, bronchoalveolar lavage, pleural fluid, and semen [14]. Here, we for the first time quantify the precise bacterial load of *U. parvum* and *U. urealyticum* using ddPCR technology. It is generally considered that *Ureaplasma* spp. load ≥10^4^ CCU/ml is a sign of active infection and may require treatment with antibiotics. The establishment of this reference value is liquid culture based. However, traditional liquid culture is only a qualitative method with relatively poor sensitivity, with false positive and false negative, as well as the inability to distinguish *U. parvum* and *U. urealyticum* [31]. Real-time PCR has become a common technique in molecular diagnosis and overcomes the above-mentioned shortcomings of liquid culture, with relative quantification through a standard curve, though the construction of a standard curve is complicated and time-consuming. Moreover, the quantitative accuracy of qPCR is easily affected by amplification efficiency and PCR inhibitors.

It’s becoming gradually accepted that role of *Ureaplasma* spp. as a pathogen in multiple diseases with increasing clinical observation studies [32, 33]. In addition, several in vitro studis have  demonstrated *that Ureaplasma* spp. modulate cytokine [34, 35] and pro-inflammatory responses in a dose-dependent manner [36]. Interestingly, one study indicated that a high load of *U. parvum* (10^7^ CFU/ml) can significantly increase prothrombin/thrombin mRNA expression to promote the onset of PPROM but that a lower load (10^5^ CFU/ml) had no significant effect [6]. It indicated that there may exist a threshold effect in various association diseases with *U. parvum* infection. Therefore, it is vital to detect the load of *Ureaplasma* spp. through more precisely quantitative technology. At this time, ddPCR, an emerging technology, is partitioned the PCR reaction into about 20,000 nm-sized droplets. Then, the relative concentration of targets, primers and probes are higher, while the inhibitor is relatively lower, which perfectly overcoming the shortcomings of conventional qPCR [23].

Our study had several limitations. First, due to the specialized instruments and consumables, ddPCR has a higher cost than qPCR, which limits its clinical application to a large extent. In addition, the prevalence of *U. urealyticum* is relatively lower and the number of populations recruited for the study was relatively small. Finally, this was a single-centre cross-sectional study, and multicenter prospective studies with a large population should be carried out to provide more evidence.

The ddPCR assay that we established has great application prospects. It can more accurately quantify the burden of *Ureaplasma* spp. than qPCR in both asymptomatic and symptomatic individuals, and monitor dynamic changes in bacterial load after the use of antibiotics. Moreover, based on its high sensitivity and high tolerance to PCR inhibitors of ddPCR, it can be used for detection of samples with low bacterial content that are readily available (urine, amniotic fluid, semen, peripheral blood, etc). Further, it can be used with chorionic amniotic tissue or lung tissue which may contain potential PCR inhibitors, promoting analysis of multiple potential diseases, such as infertility, miscarriage, chorioamnionitis, bronchopulmonary dysplasia, bacteremia, and rare invasive infections of *Ureaplasma* spp.

In conclusion, this study is the first to report the absolute quantitative analysis of *Ureaplasma* spp. with excellent analytical performance, which may be a promising technology.

## Supplementary Information

**Additional file 1. **Multiple sequence alignment of the ParC genes of 14 serotypes of *Ureaplasma* spp. (a) Sequence of the *U. parvum* primes/probe; (b) Sequence of the *U. ureaplasma* primers/probe.

## Data Availability

The data generated during the current study are available from the corresponding author upon reasonable request.

## Abbreviations

ddPCR: Digital droplet PCR
*U. parvum*: *Ureaplasma parvum*
*U. urealyticum*: *Ureaplasma urealyticum*
NSC: Non-specific cervicitis
PPROM: Preterm premature rupture of membranes
HPV: Human papillomavirus
CT: Threshold cycle
GAPDH: Glyceraldehyde-3-phosphate dehydrogenase
