# Supplementary material for: Droplet digital PCR (ddPCR) for the detection and quantification of Ureaplasma spp
Source: BMC Infect Dis. 2021 Aug 11;21:804. doi: 10.1186/s12879-021-06355-6 (PMC8359095; doi:10.1186/s12879-021-06355-6)
Supplement: Supplementary file 1 — Additional file 1. Multiple sequence alignment of the ParC genes of 14 serotypes of Ureaplasma spp. (a) Sequence of the U. parvum primes/probe; (b) Sequence of the U. ureaplasma primers/probe. [file 12879_2021_6355_MOESM1_ESM.pdf]

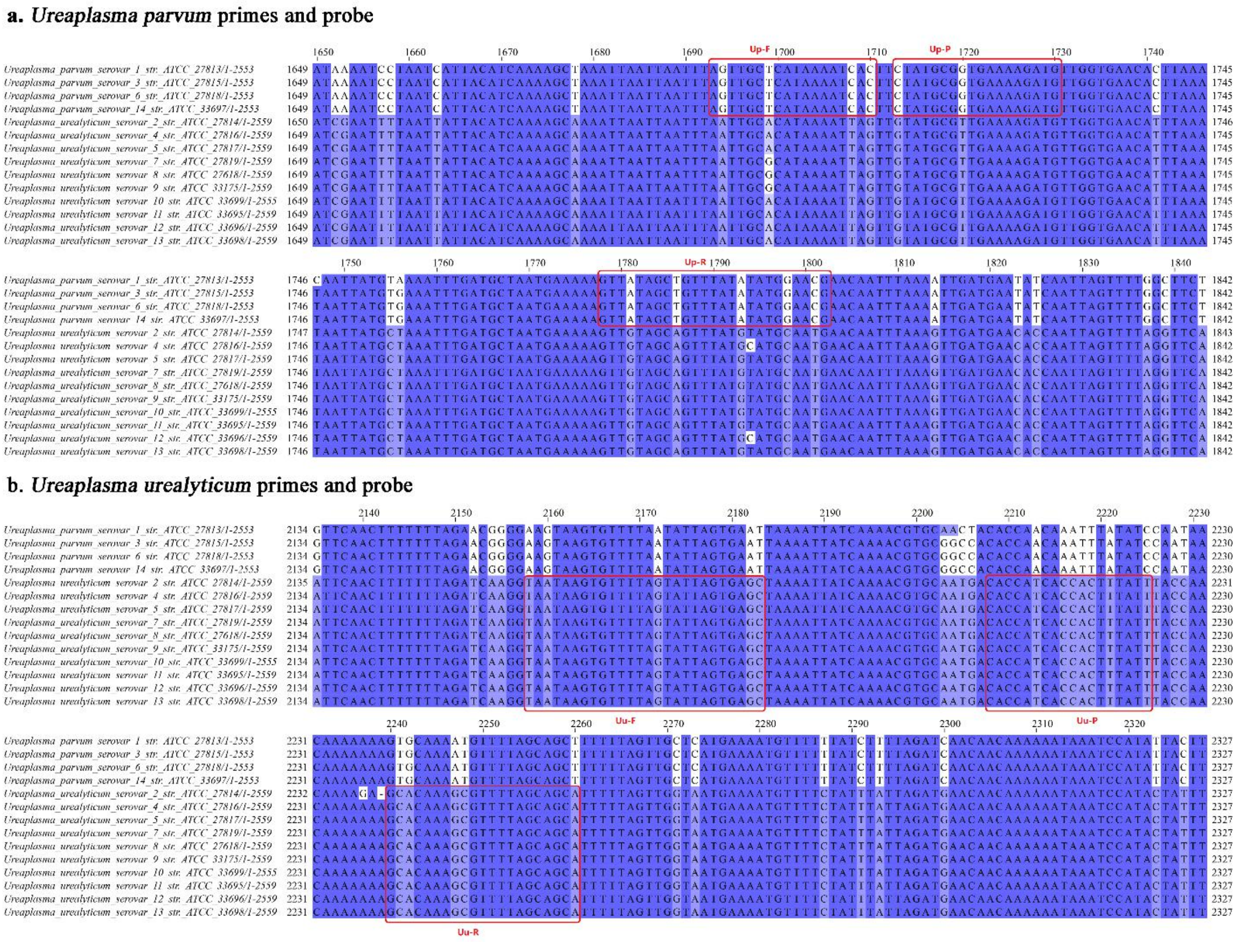

Appendix 1 Multiple sequence alignment of the ParC genes of 14 serotypes of *Ureaplasma* spp.

(a) Sequence of the *U. parvum* primers/probe; (b) Sequence of the *U. urealyticum* primers/probe.

The nucleotide sequence of 14 serovers of *Ureaplasma* spp. was downloaded from GenBank and aligned using MegaX software. The primers and TaqMan probe were designed by Primer Express 3.0 (Applied Biosystems, Foster City, CA, USA) based on conserved ParC gene regions of *U. parvum* and *U. urealyticum*. The TaqMan probes of target genes were linked to FAM at the 5’ terminus.
